# Association of M2BPGi with Subclinical Atherosclerosis in Metabolic Dysfunction-Associated Steatotic Liver Disease

**DOI:** 10.3390/metabo16080524

**Published:** 2026-07-24

**Authors:** Yong Jun Choi, Kyunghoon Lee, Han-Ik Cho, Jooheon Park, Myung Geun Shin, Ye Seol Lee, Sun Cho, Eun-Hee Nah

**Affiliations:** 1 Department of Laboratory Medicine, Chonnam National University Hwasun Hospital, Hwasun 58128, Republic of Korea; azarsis@hanmail.net (Y.J.C.); goodjusa@naver.com (J.P.); mgshin@chonnam.ac.kr (M.G.S.); 2 MEDIcheck LAB, Korea Association of Health Promotion, Seoul 07572, Republic of Korea; kama.kyunghoon@gmail.com (K.L.); hanik@snu.ac.kr (H.-I.C.); 3 MediCheck Research Institute, Korea Association of Health Promotion, Seoul 07572, Republic of Korea; yslee1270@gmail.com (Y.S.L.); dduddi3755@hanmail.net (S.C.)

**Keywords:** metabolic dysfunction-associated steatotic liver disease, cardiovascular risk, Mac-2 binding protein glycosylation isomer, hepatic fibrosis, coronary artery calcium score

## Abstract

**Background/Objectives**: Metabolic dysfunction-associated steatotic liver disease (MASLD) is increasingly recognized as a systemic metabolic disorder associated with an elevated risk of cardiovascular disease. Mac-2 binding protein glycosylation isomer (M2BPGi), a noninvasive serum biomarker of hepatic fibrosis, has also been linked to adverse metabolic and cardiovascular outcomes. However, the association between serum M2BPGi levels and subclinical coronary atherosclerosis in individuals with MASLD remains unclear. We investigated the association between serum M2BPGi levels and coronary artery calcium score (CACS), an established imaging marker of subclinical atherosclerosis, in individuals with MASLD. **Methods**: This retrospective cross-sectional study included 6514 adults with MASLD who underwent health screening examinations between 2020 and 2025. Participants were categorized into quartiles according to serum M2BPGi levels: Q1 (<0.48), Q2 (0.48–0.61), Q3 (0.62–0.80), and Q4 (≥0.81). Coronary artery calcification (CAC) was defined as CACS > 0. Multivariable logistic regression analysis was performed after adjustment for age, sex, smoking status, liver enzymes, adiposity, dysglycemia, blood pressure, and lipid profile. Multivariable ordinal logistic regression analysis was additionally performed to evaluate the association between M2BPGi levels and CAC severity. **Results**: The prevalence of CAC increased progressively across M2BPGi quartiles (39.4%, 45.3%, 48.5%, and 57.4% for Q1–Q4, respectively; *p* < 0.001). In multivariable logistic regression analysis, participants in the highest M2BPGi quartile had significantly higher odds of CAC presence than those in the lowest quartile (OR, 1.32; 95% CI, 1.10–1.58; *p* = 0.0035). Higher M2BPGi quartiles were also independently associated with greater CAC severity in multivariable ordinal logistic regression analysis (OR, 1.33; 95% CI, 1.13–1.57; *p* = 0.0007 for Q4 vs. Q1). **Conclusions**: Higher serum M2BPGi levels were independently associated with both the presence and severity of CAC in individuals with MASLD. These findings suggest that M2BPGi may serve as a potential biomarker for identifying individuals with MASLD at increased risk of subclinical atherosclerosis and may complement conventional cardiovascular risk assessment.

## 1. Introduction

Metabolic dysfunction-associated steatotic liver disease (MASLD) is the most common chronic liver disease worldwide, affecting more than one-third of the global adult population [1,2]. It is closely associated with obesity, insulin resistance, type 2 diabetes, and dyslipidemia and is increasingly recognized as a systemic metabolic disorder rather than a liver-limited disease [3].

Recent meta-analyses have established MASLD as an independent risk factor for cardiovascular disease (CVD) [4]. Beyond liver-related complications, CVD is the leading cause of death among individuals with MASLD [5]. These findings highlight the close interplay between metabolic dysfunction and cardiovascular health and underscore the importance of early cardiovascular risk assessment in this population. Accordingly, reliable strategies for identifying individuals with MASLD who are at increased cardiovascular risk are needed to optimize preventive interventions.

Accumulating evidence indicates that hepatic fibrosis, rather than simple steatosis, is the major determinant of adverse clinical outcomes in MASLD. In addition to predicting liver-related complications, advanced fibrosis has been associated with an increased risk of cardiovascular events and mortality [6]. The biological mechanisms linking hepatic fibrosis and atherosclerosis are complex and involve chronic low-grade inflammation, insulin resistance, oxidative stress, endothelial dysfunction, and extracellular matrix remodeling. These shared pathophysiological pathways suggest that biomarkers reflecting hepatic fibrogenesis may also provide information regarding cardiovascular risk.

Mac-2 binding protein glycosylation isomer (M2BPGi) has emerged as a reliable noninvasive serum biomarker for assessing hepatic fibrosis and disease progression in chronic liver diseases, including MASLD [7,8,9]. Beyond liver fibrosis, increasing evidence suggests that M2BPGi reflects systemic fibrotic activity and chronic inflammation. Experimental studies have demonstrated that M2BPGi participates in macrophage activation and extracellular matrix remodeling, both of which are involved in the development of atherosclerosis [10,11]. In addition, several clinical studies have reported positive associations between serum M2BPGi levels and cardiovascular risk factors, carotid atherosclerosis, arterial stiffness, and oxidative stress, suggesting that M2BPGi may represent a link between hepatic fibrogenesis and vascular disease [12,13,14].

Among imaging biomarkers of subclinical atherosclerosis, coronary artery calcium score (CACS) is one of the most extensively validated indicators of coronary atherosclerotic burden. Numerous prospective studies have shown that CACS independently predicts future myocardial infarction, cardiovascular mortality, and all-cause mortality beyond traditional cardiovascular risk factors [15,16]. Therefore, CACS provides an objective imaging endpoint for evaluating the relationship between emerging biomarkers and subclinical coronary atherosclerosis and is widely used for cardiovascular risk stratification in asymptomatic individuals.

Despite accumulating evidence linking MASLD with CVD and M2BPGi with hepatic fibrosis, several important knowledge gaps remain. First, most previous studies evaluating M2BPGi and CVD included relatively small populations or assessed indirect markers of atherosclerosis. Second, few studies have specifically investigated individuals with MASLD using CACS, an established imaging marker of subclinical coronary atherosclerosis. Finally, whether M2BPGi is associated not only with the presence but also with the severity of coronary artery calcification (CAC) has not been adequately investigated in a large health-screening cohort.

Therefore, using a large multicenter health-screening cohort, we investigated whether serum M2BPGi levels are independently associated with both the presence and severity of CAC in individuals with MASLD.

## 2. Materials and Methods

### 2.1. Study Population

This retrospective cross-sectional study included adults who underwent comprehensive health screening examinations between January 2020 and December 2025 at 17 health promotion centers of the Korea Association of Health Promotion. All participants voluntarily underwent standardized health screening examinations, including laboratory testing, abdominal ultrasonography (US), and coronary computed tomography (CT), according to a uniform examination protocol across all participating centers.

**Figure 1:**
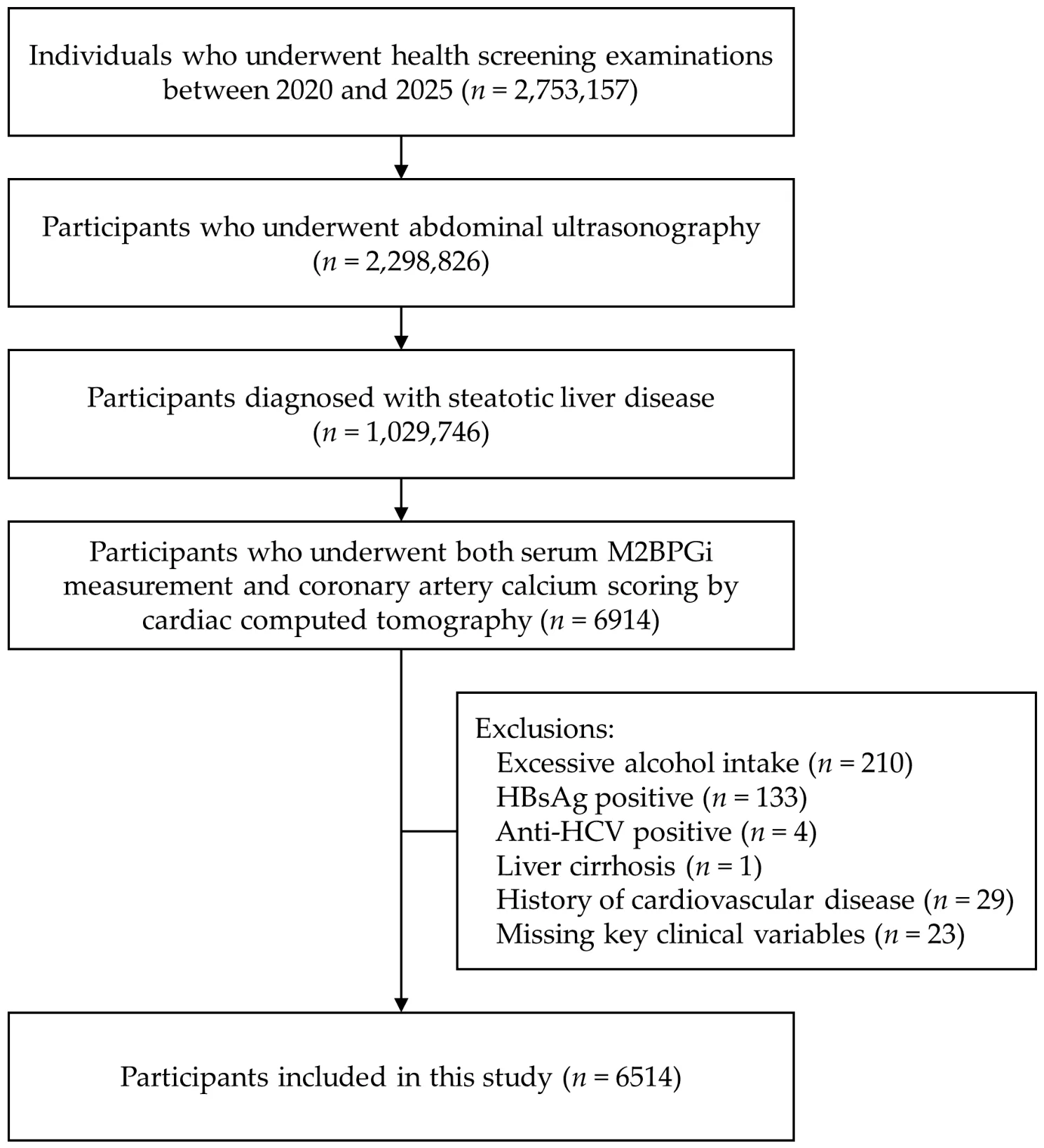
Flowchart of study population. Abbreviations: M2BPGi, Mac-2 binding protein glycosylated isomer; HBsAg, hepatitis B surface antibody; HCV, hepatitis C virus.

Individuals with available serum M2BPGi measurements and CACS were eligible for inclusion. Participants with viral hepatitis, liver cirrhosis, excessive alcohol consumption, a history of CVD, or missing key clinical variables were excluded. The detailed participant selection process is presented in Figure 1.

The study protocol was approved by the Institutional Review Board of the Korea Association of Health Promotion (IRB No. 130750-202510-HR-014). The requirement for written informed consent was waived because of the retrospective nature of the study.

### 2.2. Definition of MASLD

MASLD was defined according to the 2023 multinational consensus criteria as the presence of hepatic steatosis identified by abdominal US together with at least one cardiometabolic risk factor: (1) body mass index (BMI) ≥ 23 kg/m^2^ or waist circumference (WC) ≥ 90 cm (for males) or ≥80 cm (for females); (2) fasting blood glucose ≥ 100 mg/dL, glycated hemoglobin (HbA1c) ≥ 5.7%, type 2 diabetes, or use of glucose-lowering medication; (3) blood pressure (BP) ≥ 130/85 mmHg or use of antihypertensive medication; (4) triglycerides ≥ 150 mg/dL or use of lipid-lowering medication; or (5) HDL cholesterol < 40 mg/dL (for males) or <50 mg/dL (for females) or use of lipid-lowering medication [17].

### 2.3. Fatty Liver Assessment Using US

The presence and severity of hepatic steatosis were assessed by abdominal US. Examinations were performed by experienced radiologists or physicians trained in abdominal US using standardized imaging protocols across all participating centers. US images were obtained with the participant in the supine position and the right arm raised above the head. Hepatic steatosis was diagnosed according to established ultrasonographic criteria, including increased hepatic echogenicity, increased liver-to-kidney contrast, posterior beam attenuation, and impaired visualization of intrahepatic vessels and the diaphragm [18].

### 2.4. Laboratory Measurements

Blood samples were collected after an overnight fast of at least 8 h. Serum M2BPGi concentrations were measured using an automated chemiluminescent enzyme immunoassay system (HISCL-5000; Sysmex, Kobe, Japan) according to the manufacturer’s instructions. The assay is based on a sandwich immunoassay in which *Wisteria floribunda* agglutinin (WFA)-coated magnetic beads capture glycosylated Mac-2 binding protein (M2BP), followed by detection with an alkaline phosphatase-conjugated anti-human M2BP monoclonal antibody. Serum M2BPGi levels were expressed as the cutoff index (COI), which was calculated using the following formula: COI = ([M2BPGi] sample − [M2BPGi] negative control)/([M2BPGi] positive control − [M2BPGi] negative control) [19]. The assay was completed automatically within approximately 17 min according to the manufacturer’s protocol. Other biochemical parameters, including aspartate aminotransferase (AST), alanine aminotransferase (ALT), fasting blood glucose, HbA1c, triglycerides, and HDL cholesterol, were measured using standardized automated laboratory methods at each participating center under an internal and external quality-control program.

### 2.5. CACS Assessment

CAC was assessed using electrocardiography-gated non-contrast multidetector cardiac CT. Imaging was performed using one of the following multidetector CT scanners: Brilliance 40 (Philips Medical Systems, Cleveland, OH, USA), LightSpeed VCT 64 (GE Healthcare, Milwaukee, WI, USA), or Discovery CT750 HD (GE Healthcare, Milwaukee, WI, USA). Images were analyzed using the Extended Brilliance Workspace (Philips Medical Systems) or Advantage Workstation (GE Healthcare) by experienced readers who were blinded to the participants’ laboratory findings. CACS were calculated as described by Agatston et al. [20]. The presence of CAC was defined as CACS > 0. For the assessment of CAC severity, participants were categorized into four groups according to established clinical thresholds: CACS = 0, 1–99, 100–399, and ≥400 [21,22].

### 2.6. Statistical Analysis

All statistical analyses were performed using SAS software (version 9.4; SAS Institute, Cary, NC, USA). Continuous variables are presented as mean ± standard deviation (SD) and compared using one-way analysis of variance (ANOVA). Categorical variables are presented as frequencies and percentages and compared using the chi-square test. Missing data were handled using complete-case analysis. Linear trends across M2BPGi quartiles were assessed by assigning the mean M2BPGi value to each quartile and modeling it as a continuous variable. Multivariable logistic regression analysis was performed to evaluate the association between M2BPGi quartiles and the presence of CAC. To evaluate the association between M2BPGi and CAC severity, multivariable ordinal logistic regression analysis was performed using four ordered CAC severity categories as the dependent variable. The proportional odds assumption was evaluated using the score test before fitting the ordinal logistic regression model. Covariates were selected based on clinical relevance and previously established cardiovascular risk factors, including age (categorized as <40, 40–54, 55–69, and ≥70 years), sex, smoking status, elevated liver enzymes (AST or ALT > 40 U/L), adiposity (defined by BMI or WC), dysglycemia, BP status, hypertriglyceridemia, and low HDL cholesterol. Exploratory subgroup analyses were conducted stratified by age, sex, dysglycemia, and BP status using the same multivariable logistic regression model.

Several sensitivity analyses were performed to assess the robustness of the primary findings. First, because the association between age and CAC may be nonlinear, age was modeled using restricted cubic splines with four knots placed at the 5th, 35th, 65th, and 95th percentiles of the age distribution to provide more flexible adjustment for potential age-related confounding. The adjusted association between M2BPGi quartiles and CAC presence was reevaluated using this model, and adjusted predicted probabilities of CAC across the age range were estimated. Second, to evaluate whether the association between M2BPGi and CAC was independent of the established noninvasive hepatic fibrosis marker, the Fibrosis-4 index (FIB-4), an additional multivariable logistic regression analysis was performed. FIB-4 was calculated using age, AST, ALT, and platelet count. Because age, AST, and ALT were components of FIB-4, these variables were not entered separately into the FIB-4-adjusted model to minimize potential collinearity, whereas all other covariates remained the same as in the primary model. All statistical tests were two-sided, and a *p* value < 0.05 was considered statistically significant.

## 3. Results

### 3.1. Study Population

The study flow is illustrated in Figure 1. During the study period, 2,753,157 adults underwent comprehensive health screening examinations, of whom 2,298,826 underwent abdominal US. A total of 1,029,746 participants were identified as having steatotic liver disease. Among 6914 individuals with available serum M2BPGi measurements and CACS, 400 were excluded because of excessive alcohol consumption, viral hepatitis, liver cirrhosis, a history of CVD, or missing clinical data. Consequently, 6514 individuals with MASLD were included in the final analysis.

### 3.2. Baseline Characteristics

The baseline characteristics of the study participants according to M2BPGi quartiles are summarized in Table 1. A total of 6514 individuals with MASLD were included in the analysis. Based on serum M2BPGi levels, participants were categorized into quartiles as follows: Q1 (<0.48), Q2 (0.48–0.61), Q3 (0.62–0.80), and Q4 (≥0.81). The mean age was 57.5 ± 11.1 years, and the mean serum M2BPGi level was 0.66 ± 0.36 COI. Participants in the higher M2BPGi quartiles were progressively older, with the mean age increasing from 53.2 years in Q1 to 62.0 years in Q4 (*p* for trend < 0.001). Higher M2BPGi quartiles were also associated with significantly higher BMI, WC, systolic BP, AST, ALT, fasting blood glucose, and HbA1c levels, as well as lower HDL cholesterol levels (all *p* < 0.001). In contrast, diastolic BP and triglyceride levels did not differ significantly across the M2BPGi quartiles (*p* = 0.727 and *p* = 0.342, respectively).

### 3.3. Association Between M2BPGi and CAC and Conventional Cardiovascular Risk Factors

Table 2 summarizes the distribution of categorical clinical characteristics according to M2BPGi quartiles. Compared with participants in the lowest M2BPGi quartile, those in the highest quartile were more likely to be older, female, and to have elevated liver enzymes, adiposity, dysglycemia, high BP, hypertriglyceridemia, and low HDL cholesterol (all *p* < 0.001).

### 3.4. Association Between M2BPGi and the Presence of CAC

The mean CACS was 101.1 ± 351.0, and CAC was present in 3115 participants (47.8%). Multivariable logistic regression analysis demonstrated that higher M2BPGi quartiles were independently associated with the presence of CAC after adjustment for age, sex, smoking status, elevated liver enzymes, adiposity, dysglycemia, BP status, hypertriglyceridemia, and low HDL cholesterol (Table 3). Compared with participants in the lowest M2BPGi quartile (Q1), the adjusted ORs for CAC presence were 1.04 (95% CI, 0.87–1.25) for Q2, 1.10 (95% CI, 0.92–1.31) for Q3, and 1.32 (95% CI, 1.10–1.58) for Q4, with statistical significance observed only for Q4 (*p* = 0.0035).

Older age was the strongest independent predictor of CAC, with adjusted ORs of 3.88 (95% CI, 2.64–5.71), 13.15 (95% CI, 8.95–19.32), and 33.42 (95% CI, 21.84–51.15) for participants aged 40–54, 55–69, and ≥70 years, respectively, compared with those aged < 40 years. Current smoking (OR, 1.32; 95% CI, 1.09–1.59), former smoking (OR, 1.39; 95% CI, 1.17–1.66), prediabetes (OR, 1.20; 95% CI, 1.02–1.43), diabetes (OR, 2.28; 95% CI, 1.89–2.75), and high BP (OR, 1.69; 95% CI, 1.49–1.92) were also independently associated with CAC presence. Female sex was associated with significantly lower odds of CAC than male sex (OR, 0.39; 95% CI, 0.32–0.46), whereas elevated liver enzymes, adiposity, hypertriglyceridemia, and low HDL cholesterol were not independently associated with CAC after multivariable adjustment.

In a sensitivity analysis, age was modeled using restricted cubic splines to allow for a potential nonlinear association with CAC. After this finer adjustment for age, the association between M2BPGi and CAC was slightly attenuated. Participants in the highest M2BPGi quartile had higher estimated odds of CAC than those in the lowest quartile; however, the association did not reach statistical significance (OR, 1.20; 95% CI, 1.00–1.45; *p* = 0.0568) (Table S1). The adjusted predicted probability of CAC increased nonlinearly with age (Figure S1).

In an additional sensitivity analysis, FIB-4 was included in the multivariable model to evaluate whether the association between M2BPGi and CAC was independent of an established noninvasive fibrosis score. After adjustment for FIB-4, sex, smoking status, adiposity, dysglycemia, BP status, hypertriglyceridemia, and low HDL cholesterol, participants in the highest M2BPGi quartile remained significantly more likely to have CAC than those in the lowest quartile (OR, 1.53; 95% CI, 1.28–1.83; *p* < 0.0001). FIB-4 was also independently associated with the presence of CAC (OR, 1.96; 95% CI, 1.76–2.17; *p* < 0.0001) (Table S2).

### 3.5. Association Between M2BPGi and CAC Severity

**Figure 2:**
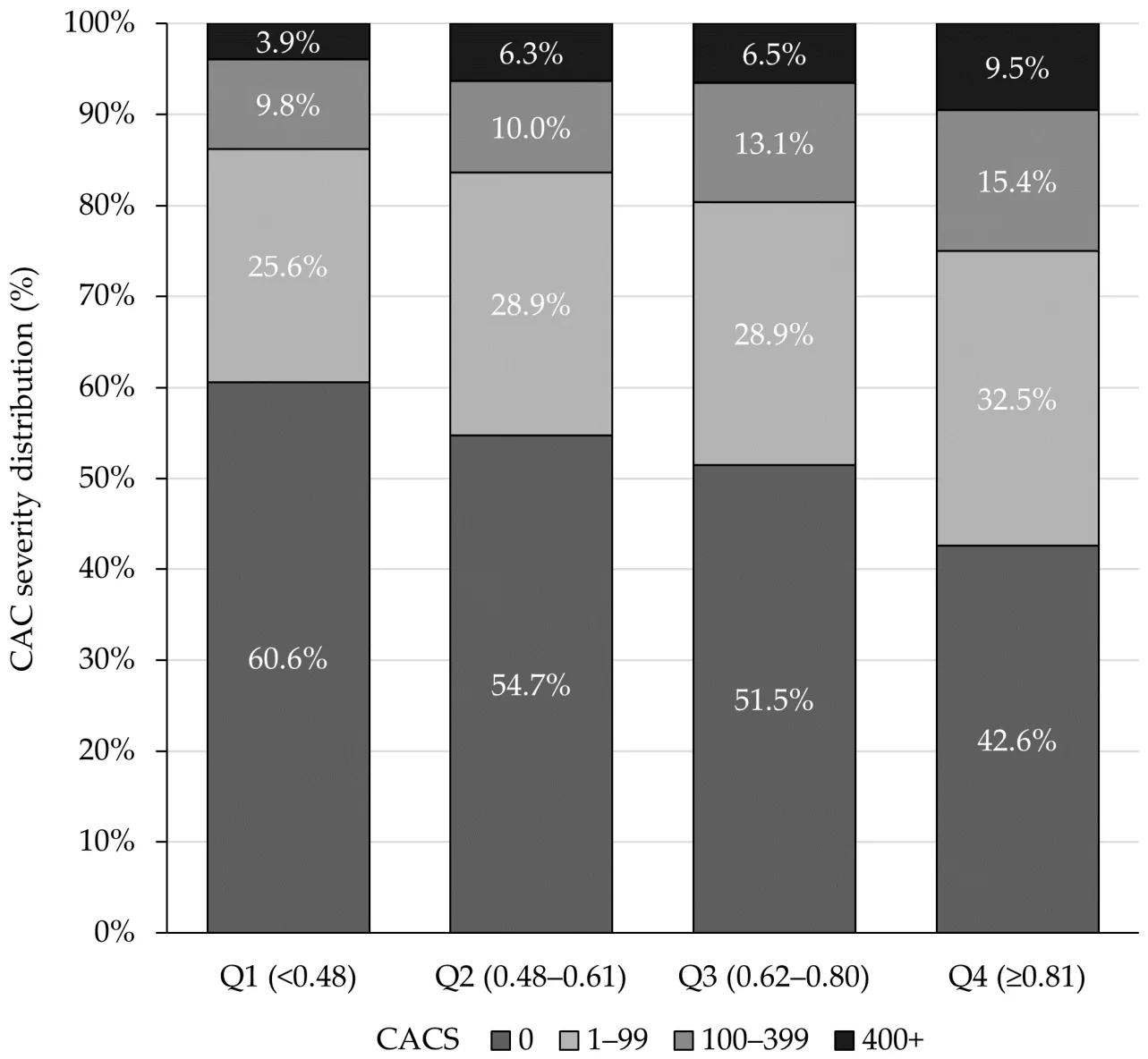
Association between M2BPGi quartiles and CAC severity. Abbreviations: M2BPGi, Mac-2 binding protein glycosylated isomer; CAC, coronary artery calcification; CACS, coronary artery calcium score.

The distribution of CAC severity according to M2BPGi quartiles is shown in Figure 2. As serum M2BPGi levels increased, the distribution of participants shifted toward more severe CAC categories. The prevalence of severe CAC (CACS ≥ 400) increased progressively from 3.9% in Q1 to 6.3%, 6.5%, and 9.5% in Q2–Q4, respectively (*p* < 0.001), whereas the proportion of participants without CAC (CACS = 0) decreased from 60.6% in Q1 to 42.6% in Q4.

To further evaluate the association between M2BPGi and CAC severity, multivariable ordinal logistic regression analysis was performed using four ordered CAC categories as the dependent variable (Table 4). Compared with participants in the lowest M2BPGi quartile (Q1), the adjusted ORs for greater CAC severity were 1.07 (95% CI, 0.91–1.26) for Q2, 1.12 (95% CI, 0.95–1.32) for Q3, and 1.33 (95% CI, 1.13–1.57) for Q4, with statistical significance observed only for the highest quartile (*p* = 0.0007). In the fully adjusted model, older age, current and former smoking, high BP, prediabetes, and diabetes were also independently associated with greater CAC severity, whereas female sex was associated with lower odds of greater CAC severity.

### 3.6. Subgroup Analysis

**Figure 3:**
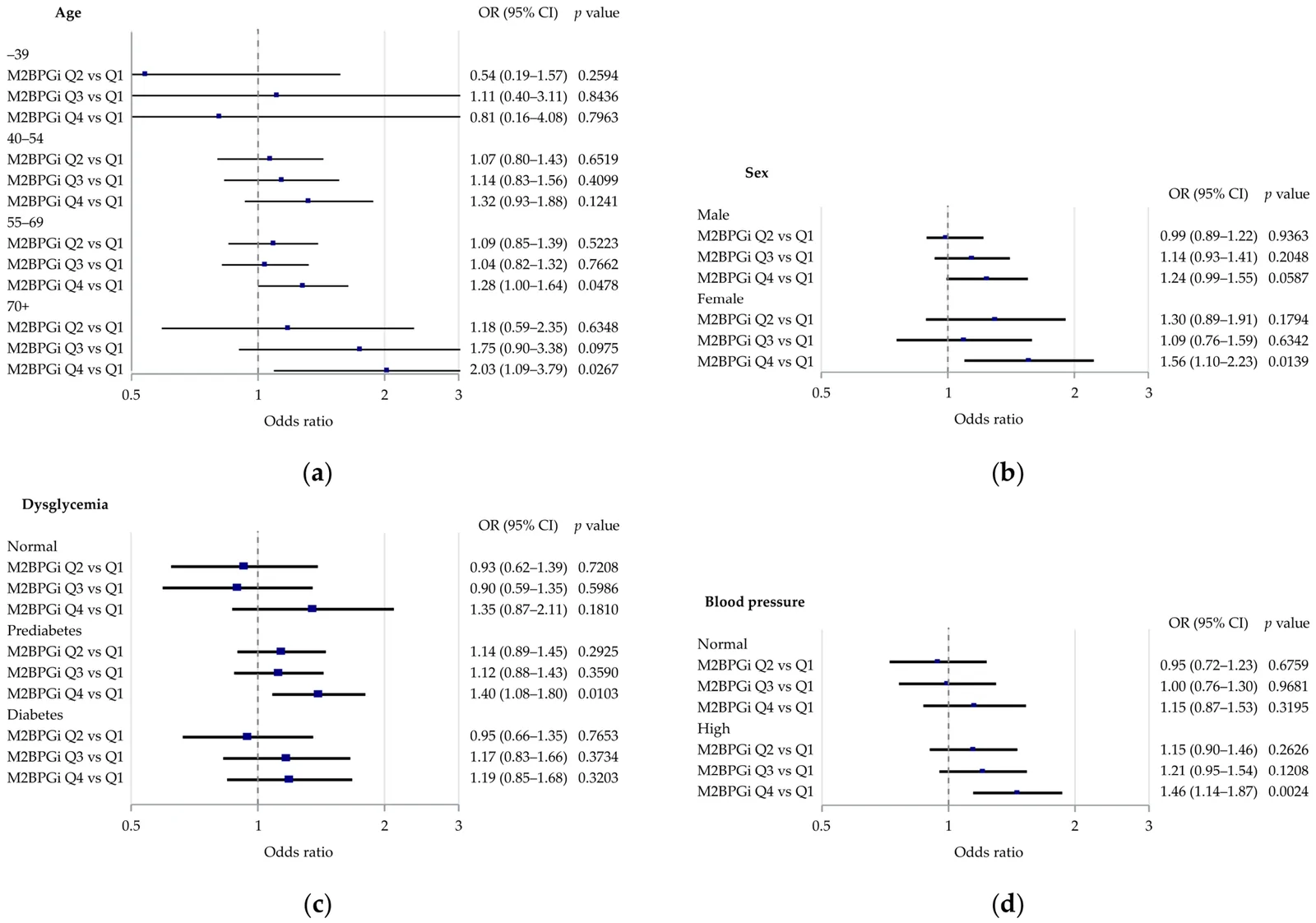
Forest plots of subgroup analyses for the association between serum M2BPGi levels and the presence of coronary artery calcification according to (**a**) age, (**b**) sex, (**c**) dysglycemia, and (**d**) blood pressure status. Abbreviations: M2BPGi, Mac-2 binding protein glycosylated isomer.

Subgroup analyses were performed to evaluate whether the association between serum M2BPGi levels and the presence of CAC differed according to age, sex, dysglycemia, and BP status (Figure 3). Compared with participants in the lowest M2BPGi quartile, those in the highest quartile had significantly higher odds of CAC presence among participants aged 55–69 years (OR, 1.28; 95% CI, 1.00–1.64; *p* = 0.0478) and ≥70 years (OR, 2.03; 95% CI, 1.09–3.79; *p* = 0.0267), female (OR, 1.56; 95% CI, 1.10–2.23; *p* = 0.0139), individuals with prediabetes (OR, 1.40; 95% CI, 1.08–1.80; *p* = 0.0103), and those with high BP (OR, 1.46; 95% CI, 1.14–1.87; *p* = 0.0024). Although these associations did not reach statistical significance in the remaining subgroups, the estimated ORs were generally in the same direction across all subgroup analyses.

## 4. Discussion

In this large cohort of individuals with MASLD, higher serum M2BPGi levels were independently associated with both the presence and severity of CAC after adjustment for conventional cardiovascular risk factors. These findings suggest that M2BPGi may reflect pathophysiological processes linking hepatic fibrosis and subclinical atherosclerosis in a health screening population.

Previous studies have demonstrated a robust association between steatotic liver disease and an increased risk of adverse cardiovascular events in the global adult population [23,24,25,26]. In addition, advanced fibrosis has emerged as the strongest hepatic predictor of adverse cardiovascular outcomes in MASLD [6]. However, the pathogenic mechanisms underlying the complex relationship between MASLD and CVD remain incompletely understood. Proposed mechanisms include visceral adiposity, chronic low-grade inflammation, oxidative stress, endothelial dysfunction, insulin resistance, dyslipidemia, intestinal dysbiosis, and genetic susceptibility [27].

Serum M2BPGi was originally developed as a biomarker for predicting hepatic fibrosis [28]. However, accumulating evidence suggests that M2BPGi may also reflect biological pathways involved in CVD. Liver fibrosis and atherosclerosis share several pathophysiological mechanisms, particularly macrophage activation and extracellular matrix remodeling. M2BPGi has been reported to function as a juxtacrine signaling mediator between hepatic stellate cells and Kupffer cells during hepatic fibrogenesis [29,30]. In addition, M2BPGi may facilitate interactions between circulating monocytes and adhesion molecules expressed on activated endothelial cells during atherosclerotic progression [31,32]. Collectively, these observations provide biological plausibility for the observed association between serum M2BPGi levels and CAC in the present study.

In our study, most conventional cardiovascular risk factors were associated with higher M2BPGi levels, suggesting that M2BPGi may reflect systemic pathophysiological processes extending beyond hepatic fibrosis. Previous studies primarily evaluated atherosclerosis using surrogate markers, such as carotid intima–media thickness or arterial stiffness, rather than CAC assessed by CT [12,13].

In contrast, subclinical atherosclerosis in the present study was assessed directly using CACS measured by multidetector CT. Higher M2BPGi levels were associated with the presence of CAC after adjustment for conventional cardiovascular risk factors. Importantly, in a sensitivity analysis including FIB-4, the association between the highest M2BPGi quartile and CAC remained statistically significant. FIB-4 itself was also independently associated with CAC presence. These findings suggest that the observed association between M2BPGi and CAC may not be fully explained by underlying hepatic fibrosis as assessed using an established noninvasive fibrosis score. However, because FIB-4 is an indirect surrogate marker rather than a direct measure of hepatic fibrosis, further studies using imaging-based or histological assessments of liver fibrosis are warranted to clarify whether M2BPGi provides cardiovascular information independent of hepatic fibrosis severity.

Interestingly, the association between M2BPGi and CAC appeared to be stronger among older individuals, females, those with prediabetes, and those with high BP in subgroup analysis. These findings should be interpreted cautiously and warrant confirmation in future prospective studies. Moreover, the prevalence of severe CAC (CACS ≥ 400) increased progressively across M2BPGi quartiles (3.9%, 6.3%, 6.5%, and 9.5% for Q1–Q4, respectively). Participants in the highest M2BPGi quartile had 33% greater odds of belonging to a more severe CAC category than those in the lowest quartile. These findings suggest that M2BPGi may reflect not only the presence but also the burden of subclinical atherosclerosis in individuals with MASLD. Although the association did not demonstrate a strictly monotonic dose–response relationship across quartiles, the overall trend supports the potential utility of M2BPGi as a practical biomarker for cardiovascular risk stratification beyond its established role as a marker of hepatic fibrosis.

This study has several limitations. First, the retrospective cross-sectional design precluded determination of causal or temporal relationships between serum M2BPGi levels and subclinical coronary atherosclerosis. Therefore, it remains unclear whether elevated M2BPGi levels precede the development of CAC or simply reflect more advanced systemic metabolic dysfunction associated with both MASLD and atherosclerosis. Although the observed associations persisted after adjustment for multiple cardiovascular risk factors, residual confounding cannot be excluded. Second, because the study population was derived from a health screening cohort, selection bias may have occurred. In addition, LDL-cholesterol measurements and detailed information on medication use, particularly statin therapy, were not consistently available across all participating centers. Although the use of antihypertensive and glucose-lowering medications was incorporated into the definitions of high BP and diabetes, respectively, residual confounding related to lipid levels and medication use cannot be excluded. Third, hepatic steatosis was assessed using US. Although US is a practical, noninvasive, and widely used method for detecting hepatic steatosis, it has lower sensitivity for mild steatosis than liver biopsy or magnetic resonance-based techniques. Therefore, some individuals with mild hepatic steatosis may have been misclassified [33]. Furthermore, ultrasonographic steatosis grade could not be included in the present analysis because ultrasonographic steatosis grade was not consistently available across all participating centers. Fourth, although the association between M2BPGi and CAC remained significant after adjustment for FIB-4, direct measures of hepatic fibrosis, such as magnetic resonance elastography or liver biopsy, were not available for the entire study population. Therefore, residual confounding by hepatic fibrosis severity cannot be completely excluded.

Despite these limitations, this study has several strengths. First, this study included a large nationwide sample from 17 health promotion centers and integrated participants who underwent both serum M2BPGi measurement and coronary CT evaluation, enabling a comprehensive assessment of the relationship between M2BPGi and cardiovascular risk. To our knowledge, this is one of the largest studies to investigate the association between M2BPGi and CAC in individuals with MASLD. Second, this study demonstrated associations between M2BPGi and not only the presence but also the severity of subclinical atherosclerosis. Given its accessibility and ease of measurement, M2BPGi may complement conventional cardiovascular risk assessment in individuals with MASLD. Further prospective studies are warranted to determine whether incorporation of M2BPGi into existing cardiovascular risk prediction models improves identification of individuals at high cardiovascular risk.

## 5. Conclusions

Higher serum M2BPGi levels were associated with a greater prevalence and severity of CAC in individuals with MASLD, although the association with CAC presence was attenuated after more refined adjustment for age. These findings suggest a potential relationship between M2BPGi and subclinical coronary atherosclerosis; however, the cross-sectional design precludes causal or temporal inferences. Further longitudinal studies are needed to determine whether M2BPGi predicts the development or progression of CAC and improves cardiovascular risk stratification in individuals with MASLD.

## Figures and Tables

**Table 1:** Baseline characteristics of study participants according to M2BPGi quartiles.

Characteristic	*n*	M2BPGi				*p* Value
		Q1 (<0.48)	Q2 (0.48–0.61)	Q3 (0.62–0.80)	Q4 (≥0.81)	
Age, year	6514	53.15 (10.83)	56.36 (10.66)	58.16 (10.64)	61.97 (10.40)	<0.0001
AST, U/L	6514	31.11 (14.80)	33.42 (17.06)	36.37 (27.33)	39.40 (26.10)	<0.0001
ALT, U/L	6514	34.53 (22.44)	37.13 (26.21)	38.97 (28.73)	40.47 (30.11)	<0.0001
Body mass index, kg/m^2^	6514	26.05 (3.35)	26.34 (3.28)	26.60 (3.69)	26.64 (3.45)	<0.0001
Waist circumference, cm	6505	89.11 (8.65)	89.96 (8.74)	90.10 (9.54)	90.36 (8.76)	0.0005
Blood glucose, mg/dL	6501	105.89 (20.43)	110.14 (23.48)	112.87 (27.48)	118.87 (35.37)	<0.0001
Glycated hemoglobin, %	5674	5.96 (0.80)	6.10 (0.93)	6.17 (0.99)	6.39 (1.27)	<0.0001
Systolic BP, mmHg	6510	120.79 (13.70)	121.79 (13.57)	122.32 (13.71)	123.54 (14.18)	<0.0001
Diastolic BP, mmHg	6510	76.14 (9.69)	76.35 (9.24)	76.32 (9.34)	76.52 (9.33)	0.7269
Triglycerides, mg/dL	6404	139.38 (91.14)	156.89 (111.00)	163.73 (116.63)	164.49 (131.62)	0.3416
HDL cholesterol, mg/dL	6404	50.46 (11.71)	49.64 (11.72)	48.95 (11.77)	48.36 (12.55)	<0.0001

Data are mean (standard deviation) values. *p* values are from one-way analysis of variance (ANOVA) comparing the mean values of the outcome across the M2BPGi quartiles. Abbreviations: M2BPGi, Mac-2 binding protein glycosylated isomer; AST, aspartate aminotransferase; ALT, alanine aminotransferase; BP, blood pressure.

**Table 2:** Distribution of categorical clinical characteristics according to M2BPGi quartiles.

Risk Factors	Total	M2BPGi				*p* Value
		Q1 (<0.48)	Q2 (0.48–0.61)	Q3 (0.62–0.80)	Q4 (≥0.81)	
Total	6514 (100.0%)	1579 (24.2%)	1591 (24.4%)	1655 (25.4%)	1689 (25.9%)	
CAC						<0.0001
Absence	3399 (52.2%)	957 (60.6%)	871 (54.7%)	852 (51.5%)	719 (42.6%)	
Presence	3115 (47.8%)	622 (39.4%)	720 (45.3%)	803 (48.5%)	970 (57.4%)	
CACS						<0.0001
0	3399 (52.2%)	957 (60.6%)	871 (54.7%)	852 (51.5%)	719 (42.6%)	
1–99	1893 (29.1%)	405 (25.6%)	460 (28.9%)	479 (28.9%)	549 (32.5%)	
100–399	790 (12.1%)	155 (9.8%)	159 (10.0%)	216 (13.1%)	260 (15.4%)	
400+	432 (6.6%)	62 (3.9%)	101 (6.3%)	108 (6.5%)	161 (9.5%)	
Age, year						<0.0001
1–39	439 (6.7%)	186 (11.8%)	117 (7.4%)	92 (5.6%)	44 (2.6%)	
40–54	1934 (29.7%)	635 (40.2%)	526 (33.1%)	455 (27.5%)	318 (18.8%)	
55–69	3292 (50.5%)	683 (43.3%)	781 (49.1%)	890 (53.8%)	938 (55.5%)	
70+	849 (13.0%)	75 (4.7%)	167 (10.5%)	218 (13.2%)	389 (23.0%)	
Sex						<0.0001
Male	4482 (68.8%)	1262 (79.9%)	1150 (72.3%)	1110 (67.1%)	960 (56.8%)	
Female	2032 (31.2%)	317 (20.1%)	441 (27.7%)	545 (32.9%)	729 (43.2%)	
Smoking						<0.0001
Never	2557 (39.3%)	553 (35.0%)	576 (36.2%)	659 (39.8%)	769 (45.5%)	
Current	1111 (17.1%)	309 (19.6%)	302 (19.0%)	262 (15.8%)	238 (14.1%)	
Former	1570 (24.1%)	450 (28.5%)	387 (24.3%)	397 (24.0%)	336 (19.9%)	
Missing	1276 (19.6%)	267 (16.9%)	326 (20.5%)	337 (20.4%)	346 (20.5%)	
AST or ALT						<0.0001
Normal (≤40U/L)	5339 (82.0%)	1400 (88.7%)	1343 (84.4%)	1319 (79.7%)	1277 (75.6%)	
Elevated (>40U/L)	1175 (18.0%)	179 (11.3%)	248 (15.6%)	336 (20.3%)	412 (24.4%)	
BMI or WC						0.0001
Normal	768 (11.8%)	233 (14.8%)	177 (11.1%)	192 (11.6%)	166 (9.8%)	
High	5746 (88.2%)	1346 (85.2%)	1414 (88.9%)	1463 (88.4%)	1523 (90.2%)	
Dysglycemia						<0.0001
Normal	1314 (20.2%)	418 (26.5%)	331 (20.8%)	307 (18.6%)	258 (15.3%)	
Prediabetes	3300 (50.7%)	838 (53.1%)	833 (52.4%)	836 (50.6%)	793 (47.1%)	
Diabetes	1890 (29.1%)	323 (20.5%)	426 (26.8%)	508 (30.8%)	633 (37.6%)	
Blood pressure						<0.0001
Normal	3158 (48.5%)	848 (53.7%)	775 (48.7%)	812 (49.1%)	723 (42.9%)	
High	3352 (51.5%)	731 (46.3%)	816 (51.3%)	842 (50.9%)	963 (57.1%)	
Triglyceride						0.0003
Normal	3170 (49.0%)	848 (53.8%)	773 (48.8%)	762 (46.6%)	787 (47.1%)	
High	3296 (51.0%)	727 (46.2%)	810 (51.2%)	874 (53.4%)	885 (52.9%)	
HDL cholesterol						<0.0001
Normal	3593 (55.6%)	1001 (63.6%)	906 (57.2%)	896 (54.8%)	790 (47.2%)	
Low	2873 (44.4%)	574 (36.4%)	677 (42.8%)	740 (45.2%)	882 (52.8%)	

Data are frequency (percentage) values. *p* values are from a chi-square test comparing the frequencies of the outcomes across M2BPGi quartiles. Abbreviations: M2BPGi, Mac-2 binding protein glycosylated isomer; CAC, coronary artery calcification; CACS, coronary artery calcium score; AST, aspartate aminotransferase; ALT, alanine aminotransferase; BMI, body mass index; WC, waist circumference.

**Table 3:** Multivariable logistic regression analysis for the presence of coronary artery calcification.

Risk Factors	OR	95% CI	*p* Value
M2BPGi			
Q1 (<0.48)	ref.		
Q2 (0.48–0.61)	1.04	0.87–1.25	0.6523
Q3 (0.62–0.80)	1.10	0.92–1.31	0.3128
Q4 (≥0.81)	1.32	1.10–1.58	0.0035
Age, year			
1–39	ref.		
40–54	3.88	2.64–5.71	<0.0001
55–69	13.15	8.95–19.32	<0.0001
70+	33.42	21.84–51.15	<0.0001
Sex			
Male	ref.		
Female	0.39	0.32–0.46	<0.0001
Smoking			
Never	ref.		
Current	1.32	1.09–1.59	0.0041
Former	1.39	1.17–1.66	0.0002
AST or ALT			
Normal (≤40U/L)	ref.		
Elevated (>40U/L)	1.00	0.85–1.18	0.9618
BMI or WC			
Normal	ref.		
High	1.08	0.89–1.31	0.4566
Dysglycemia			
Normal	ref.		
Prediabetes	1.20	1.02–1.43	0.0305
Diabetes	2.28	1.89–2.75	<0.0001
Blood pressure			
Normal	ref.		
High	1.69	1.49–1.92	<0.0001
Triglyceride			
Normal	ref.		
High	1.02	0.89–1.18	0.7525
HDL cholesterol			
Normal	ref.		
Low	1.04	0.90–1.20	0.5702

Abbreviations: M2BPGi, Mac-2 binding protein glycosylated isomer; AST, aspartate aminotransferase; ALT, alanine aminotransferase; BMI, body mass index; WC, waist circumference.

**Table 4:** Association between M2BPGi and coronary artery calcification severity assessed by ordinal logistic regression.

M2BPGi	OR	95% CI	*p* Value
Q1 (<0.48)	ref.		
Q2 (0.48–0.61)	1.07	0.91–1.26	0.4163
Q3 (0.62–0.80)	1.12	0.95–1.32	0.1765
Q4 (≥0.81)	1.33	1.13–1.57	0.0007

Adjusted for age, sex, smoking status, elevated liver enzymes, adiposity, dysglycemia, blood pressure status, hypertriglyceridemia, and low HDL cholesterol. Abbreviations: M2BPGi, Mac-2 binding protein glycosylated isomer.

## Data Availability

The data presented in this study are available upon request from the corresponding author upon reasonable request due to institutional ethical restrictions.

## Supplementary Materials

The following supporting information can be downloaded at https://www.mdpi.com/article/10.3390/metabo16080524/s1, Table S1: Sensitivity analysis of the association between M2BPGi levels and CAC presence using restricted cubic splines for age; Figure S1: Adjusted predicted probability of CAC according to age using restricted cubic spline analysis; Table S2: Sensitivity analysis of the association between M2BPGi levels and CAC presence after adjustment for FIB-4.
