# Supplementary material for: Association of M2BPGi with Subclinical Atherosclerosis in Metabolic Dysfunction-Associated Steatotic Liver Disease
Source: Metabolites. 2026 Jul 24;16(8):524. doi: 10.3390/metabo16080524 (PMC13515549; doi:10.3390/metabo16080524)
Supplement: Supplementary file 1 [file metabolites-16-00524-s001.zip › metabolites-4428096-supplementary.pdf]

Table S1. Sensitivity analysis of the association between M2BPGi levels and CAC presence using restricted cubic splines for age.

Risk Factors	OR	95% CI		*p* Value
M2BPGi				
Q1 (<0.48)	ref.			
Q2 (0.48–0.61)	0.99	0.83	1.19	0.9426
Q3 (0.62–0.80)	1.04	0.87	1.25	0.6585
Q4 (≥0.81)	1.20	1.00	1.45	0.0568
Age (restricted cubic spline)				<0.0001
Sex				
Male	ref.			
Female	0.37	0.31	0.44	<0.0001
Smoking				
Never	ref.			
Current	1.35	1.12	1.63	0.0021
Former	1.38	1.16	1.65	0.0003
AST or ALT				
Normal (≤40U/L)	ref.			
Elevated (>40U/L)	1.04	0.88	1.22	0.6868
BMI or WC				
Normal	ref.			
High	1.15	0.94	1.40	0.1755
Dysglycemia				
Normal	ref.			
Prediabetes	1.16	0.98	1.38	0.0829
Diabetes	2.17	1.80	2.62	<0.0001
Blood pressure				
Normal	ref.			
High	1.62	1.42	1.84	<0.0001
Triglyceride				
Normal	ref.			
High	1.07	0.93	1.23	0.3440
HDL-cholesterol				
Normal	ref.			
Low	1.03	0.89	1.19	0.7115

Age was modeled using a restricted cubic spline; therefore, a single OR and 95% CI are not presented. Abbreviations: M2BPGi, Mac-2-binding protein glycosylated isomer; CAC, coronary artery calcification; AST, aspartate aminotransferase; ALT, alanine aminotransferase; BMI, body mass index; WC, waist circumference.

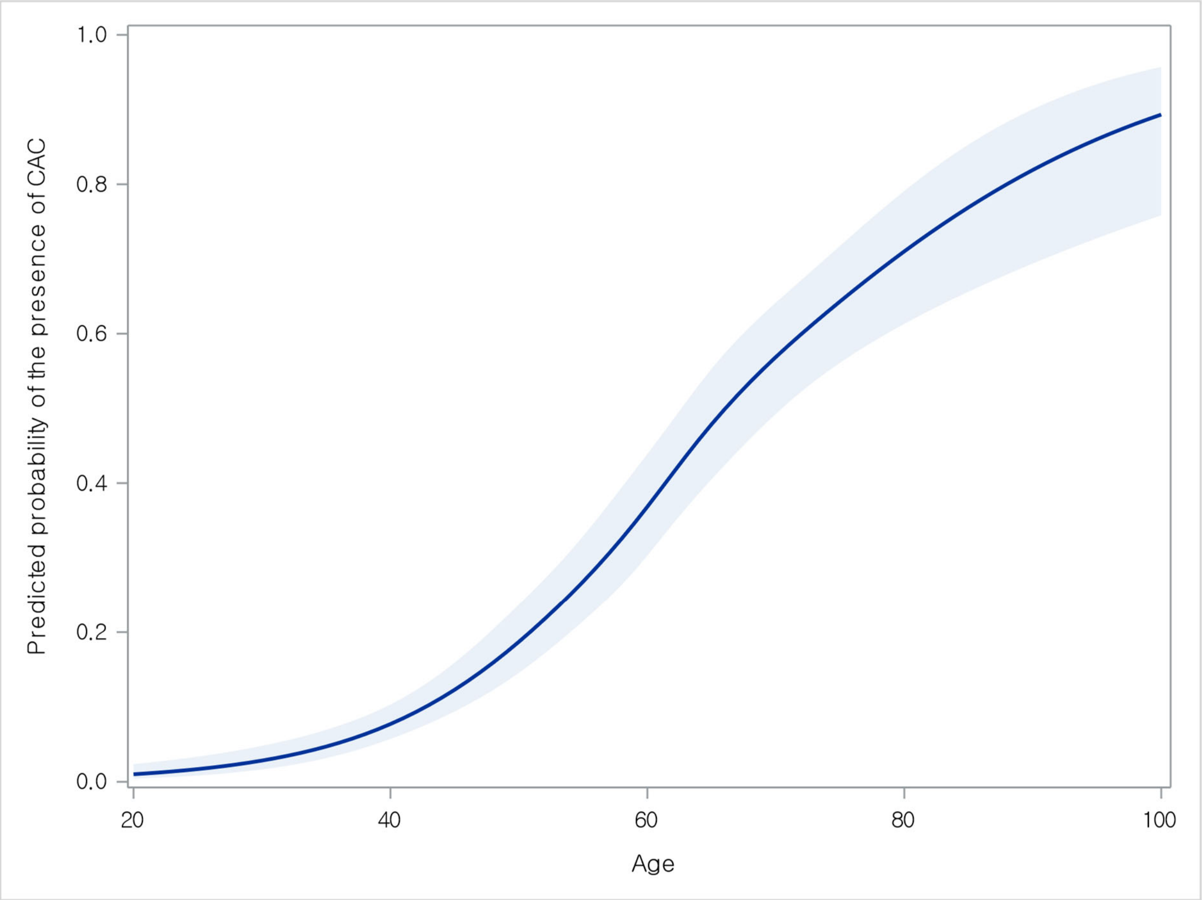

Figure S1. Adjusted predicted probability of CAC according to age using restricted cubic spline analysis. Age was modeled using restricted cubic splines with four knots placed at the 5th, 35th, 65th, and 95th percentiles of the age distribution. The solid line represents the adjusted predicted probability of CAC, and the shaded area represents the 95% CI. Abbreviations: CAC, coronary artery calcification.

Table S2. Sensitivity analysis of the association between M2BPGi levels and CAC presence after adjustment for FIB-4.

Risk Factors	OR	95% CI		*p* Value
M2BPGi				
Q1 (<0.48)	ref.			
Q2 (0.48–0.61)	1.11	0.94	1.32	0.2320
Q3 (0.62–0.80)	1.26	1.06	1.50	0.0080
Q4 (≥0.81)	1.53	1.28	1.83	<0.0001
FIB-4	1.96	1.76	2.17	<0.0001
Sex				
Male	ref.			
Female	0.57	0.48	0.67	<0.0001
Smoking				
Never	ref.			
Current	1.22	1.03	1.46	0.0259
Former	1.49	1.26	1.76	<0.0001
BMI or WC				
Normal	ref.			
High	0.92	0.76	1.12	0.3985
Dysglycemia				
Normal	ref.			
Prediabetes	1.45	1.23	1.70	<0.0001
Diabetes	2.88	2.40	3.45	<0.0001
Blood pressure				
Normal	ref.			
High	1.91	1.69	2.16	<0.0001
Triglyceride				
Normal	ref.			
High	0.92	0.80	1.06	0.2386
HDL-cholesterol				
Normal	ref.			
Low	1.19	1.03	1.36	0.0147

Abbreviations: Mac-2-binding protein glycosylated isomer; CAC, coronary artery calcification; FIB-4, Fibrosis-4 index; BMI, Body mass index; WC, Waist circumference.
